# RETRACTION: The DNMT1/miR‐34a Axis Is Involved in the Stemness of Human Osteosarcoma Cells and Derived Stem‐Like Cells

**DOI:** 10.1155/sci/9767394

**Published:** 2026-03-26

**Authors:** Stem Cells International

RETRACTION: X. Liang, C. Xu, and W. Wang, X. Li, “The DNMT1/miR‐34a Axis Is Involved in the Stemness of Human Osteosarcoma Cells and Derived Stem‐Like Cells,” *Stem Cells International*, 2019, 7028901, https://doi.org/10.1155/2019/7028901.

The above article, published online on 31 October 2019 in Wiley Online Library (wileyonlinelibrary.com), has been retracted by agreement between the journal Associate Editor, Prof. James Adjaye; and John Wiley & Sons Ltd. The retraction has been agreed due to multiple inconsistencies in the figures, initially identified on PubPeer [1]. Further investigation identified additional concerns. A summary of all figure concerns is as follows:

• The CD44 blots in Figure 1h and Oct4 blots in Figure 1i appear identical
• The ß‐actin blots in Figure 1h, the DNMT1 blots in Figure 6d, and ß‐actin blots in fourth row of Figure 6g appear identical
• The CD44 blots in Figure 1h and Oct4 blots in Figure 1i, appear similar to:
  ⚬ The CD44 blots in Figure 1b of [2]
  ⚬ The pERK1/2 blots in Figure 4a of [3]
• The ß‐actin blots in Figure 1i appear similar to the ß‐actin blots shown in Figure 2h of [4]
• The CD133 blots in Figure 1h appear similar to the P2/ABCG1 blots shown in Figure 1a of [5]
• The ABCG2 blots in Figure 1h appear similar to the ABCG1 blots shown in Figure 2a of [5]
• The OSLC image in Figure 2e appears identical to the lefthand mouse image in Figure 1i of [6]
• In Figure 3h, the final three CD133 bands (1‐5µm) appear identical to the first three Bmi1 bands shown in Figure 3i (0‐2.5µm)
• In Figure 3h, the final three CD44 bands (1‐5µm) appear identical to the first three Sox2 blots shown in Figure 3i (0‐2.5µm)
• The 5µm Aza‐dC panel shown in Figure 3f appears to be identical to the Ad‐GFP+ image shown in Figure 3g of [7]
• The CD44 bands shown in Figure 3h appear similar to the DNMT1 bands shown in Figure 2j of [8]
• The first three Sox2 bands (0‐2.5µm) shown in Figure 3h appear similar to the final three DNMT1 bands (10‐40mg/kg) shown in Figure 2j of [8]
• The second and third ß‐actin bands (shNC and shDNMT1) shown in the second row of Figure 4f appear to be identical to the first and second ß‐actin bands (OSLC and shNC) shown in the second row of Figure 4f
• The final row of ß‐actin bands shown in Figures 4g and 5g appear identical
• The Bmi1 bands shown in Figure 4g appear similar to the FoxM1 bands shown in Figure 7d of [6]
• The ß‐actin bands shown in Figure 5c appear similar to:
  ⚬ The H292/actin bands shown in Figure 6a of [9]
  ⚬ The actin bands shown in Figure 2c of [9]
  ⚬ The STAT3 bands shown in Figure 5c of [10]
• The first row of ß‐actin bands shown in Figure 5g appear similar to the JAK2 bands shown in Figure 3f of [11]
• The second row of ß‐actin bands shown in Figure 5g appear similar to the ß‐actin bands shown in Figure 2e of [12]
• The CD44 bands shown in Figure 5f appear to be similar to the H3K14 bands shown in Figure 3a of [13]
• The CD133 bands shown in Figure 5f appear to be similar to the ATF5 bands shown in Figure 3a of [13]
• The DNMT1 bands shown in Figure 6d appear similar to the fourth row (ß‐actin) bands shown in Figure 6g
• The first ß‐actin band in the fourth and sixth rows of Figure 6h are identical
• The CD133 bands shown in Figure 6g appear similar to the p‐Akt bands shown in Figure 2d of [14]
• The Sox2 bands shown in Figure 6h appear identical to the CD133 bands shown in Figure 4d of [7]
• The ß‐actin bands shown in the fourth row of Figure 6g appear similar to the actin bands shown in Figure 7c of [15]
• The ß‐actin bands shown in the fourth row of Figure 7g appear similar to the ß‐actin bands shown in Figure 5j of [16]
• The Bmi1 bands shown in Figure 7h appear similar to the Bmi1 bands shown in Figure 2i of [7]
• The Oct4 bands shown in Figure 7h appear similar to the E‐cadherin bands shown in Figure 3d of [16]

These concerns have resulted in the editorial board and publisher losing confidence in the published results. Therefore, the article is retracted.

The authors did not respond to indicate their agreement to the retraction.

## References

[1] biskrensis A. , camphorifolia H. , and arachidis M. , The DNMT1/miR-34a Axis Is Involved in the Stemness of Human Osteosarcoma Cells and Derived Stem-Like Cells, *PubPeer*, 2022 https://pubpeer.com/publications/C1DB550329F43AA003ACAFEBAA69AF. 10.1155/2019/7028901 PMC6875320 31781245

[2] Wen Q. , Xu C. , and Zhou J. , et al.RETRACTED ARTICLE: 8-Bromo-7-Methoxychrysin Suppress Stemness of SMMC-7721 Cells Induced by Co-Culture of Liver Cancer Stem-Like Cells With Hepatic Stellate Cells, BMC Cancer. (2019) 19, no. 1, 224 10.1186/s12885-019-5419-5, 2-s2.0-85062843000. PMC6416872 30866863

[3] Wang X. Q. , Lo C. M. , Chen L. , Ngan E. S.-W. , Xu A. , and Poon R. Y. C. , CDK1-PDK1-PI3K/Akt Signaling Pathway Regulates Embryonic and Induced Pluripotency, Cell Death & Differentiation. (2017) 24, no. 1, 38–48, 10.1038/cdd.2016.84, 2-s2.0-84988354816. 27636107 PMC5260505

[4] Fu Z. , Cao X. , Yang Y. , Song Z. , Zhang J. , and Wang Z. , Upregulation of FoxM1 by MnSOD Overexpression Contributes to Cancer Stem-Like Cell Characteristics in the Lung Cancer H460 Cell Line, Technology in Cancer Research & Treatment. (2018) 17, no. 1, 1–12, 10.1177/1533033818789635, 2-s2.0-85056698149. PMC6096686 30111255

[5] Tian C. , Huang D. , Yu Y. , Zhang J. , Fang Q. , and Xie C. , ABCG1 as a Potential Oncogene in Lung Cancer, Experimental and Therapeutic Medicine. (2017) 13, no. 6, 3189–3194, 10.3892/etm.2017.4393, 2-s2.0-85019115262. 28588672 PMC5450787

[6] Cao X. , Liu L. , and Cao X. , et al.The DNMT1/miR-34a/FOXM1 Axis Contributes to Stemness of Liver Cancer Cells, Journal of Oncology. (2020) 2020, 10.1155/2020/8978930, 8978930. 32308683 PMC7142390

[7] Cao X. , Liu L. , and Yuan Q. , et al.RETRACTED ARTICLE: Isovitexin Reduces Carcinogenicity and Stemness in Hepatic Carcinoma Stem-Like Cells by Modulating MnSOD and FoxM1, Journal of Experimental & Clinical Cancer Research. (2019) 38, no. 1, 264 10.1186/s13046-019-1244-6, 2-s2.0-85067553671. PMC6580799 31208440

[8] Liang X. , Xu C. , Cao X. , and Wang W. , Isovitexin Suppresses Cancer Stemness Property and Induces Apoptosis of Osteosarcoma Cells by Disruption of the DNMT1/miR-34a/Bcl-2 Axis, Cancer Management and Research. (2019) 11, no. 1, 8923–8936, 10.2147/CMAR.S222708, 2-s2.0-85074149214. 31686915 PMC6800563

[9] Amin A. R. , Haque A. , and Rahman M. A. , et al.Curcumin Induces Apoptosis of Upper Aerodigestive Tract Cancer Cells by Targeting Multiple Pathways, PLoS ONE. (2015) 10, no. 4, 10.1371/journal.pone.0124218, 2-s2.0-84929261513, e0124218. 25910231 PMC4409383

[10] Zhang Y. , Chen F. , Xiao X. , Pan W. , Yuan Q. , and Cao J. , Chrysin Inhibits Sphere Formation in SMMC-7721 Cells via Modulation of SHP-1/STAT3 Signaling Pathway, Cancer Management and Research. (2019) 11, no. 1, 2977–2985, 10.2147/CMAR.S193647, 2-s2.0-85065072275. 31114345 PMC6497861

[11] Kim S. , Gwak H. R. , Kim H. S. , Kim B. , Dhanasekaran D. N. , and Song Y. S. , Malignant Ascites Enhances Migratory and Invasive Properties of Ovarian Cancer Cells With Membrane Bound IL-6R *in vitro* , Oncotarget. (2016) 7, no. 50, 83148–83159, 10.18632/oncotarget.13074, 2-s2.0-85003967088. 27825119 PMC5347759

[12] Liu F. , Yuan Q. , and Cao X. , et al.RETRACTED: Isovitexin Suppresses Stemness of Lung Cancer Stem-Like Cells Through Blockage of MnSOD/CaMKII/AMPK Signaling and Glycolysis Inhibition, BioMed Research International. (2021) 2021, 10.1155/2021/9972057, 9972057. 34195288 PMC8203360

[13] Huang R. , Qian D. , and Hu M. , et al.Association Between Human Cytomegalovirus Infection and Histone Acetylation Level in Various Histological Types of Glioma, Oncology Letters. (2015) 10, no. 5, 2812–2820, 10.3892/ol.2015.3638, 2-s2.0-84942416370. 26722247 PMC4665835

[14] Chen W. , Wu J. , and Shi H. , et al.Hepatic Stellate Cell Coculture Enables Sorafenib Resistance in Huh7 Cells Through HGF/c-Met/Akt and Jak2/Stat3 Pathways, BioMed Research International. (2014) 2014, 10.1155/2014/764981, 2-s2.0-84904112646, 764981. 25057499 PMC4095710

[15] Guan F. , Ding Y. , and Zhang Y. , et al.Curcumin Suppresses Proliferation and Migration of MDA-MB-231 Breast Cancer Cells Through Autophagy-Dependent Akt Degradation, PLoS ONE. (2016) 11, no. 1, 10.1371/journal.pone.0146553, 2-s2.0-84954567599, e0146553. 26752181 PMC4708990

[16] Qiu Y. , Cao X. , and Liu L. , et al.Modulation of MnSOD and FoxM1 Is Involved in Invasion and EMT Suppression by Isovitexin in Hepatocellular Carcinoma Cells, Cancer Management and Research. (2020) 12, no. 1, 5759–5771, 10.2147/CMAR.S245283. 32765079 PMC7371559

